# GP73 Is Upregulated by Hepatitis C Virus (HCV) Infection and Enhances HCV Secretion

**DOI:** 10.1371/journal.pone.0090553

**Published:** 2014-03-07

**Authors:** Longbo Hu, Wenxia Yao, Fang Wang, Xia Rong, Tao Peng

**Affiliations:** 1 State Key Laboratory of Respiratory Diseases, Guangzhou Institute of Biomedicine and Health, Chinese Academy of Sciences, Guangzhou, China; 2 Guangzhou Blood Center, Guangzhou, China; 3 South China United Vaccine Insititute, Guangzhou, China; Institute of Molecular and Cell Biology, Biopolis, United States of America

## Abstract

Hepatitis C virus (HCV) is a major cause of chronic liver disease. However, little is known about the details of its assembly and secretion. Golgi-related proteins have been recently proven to have a key function in HCV secretion. Golgi protein 73 (GP73), a resident Golgi membrane protein, is a potential serum biomarker for the diagnosis of liver diseases and hepatocellular carcinoma. Previous studies have demonstrated the upregulation of GP73 in the liver samples and sera of HCV-infected patients. However, the function and regulatory mechanism of GP73 in HCV infection at the cellular level remain unknown. In this study, we examined the expression level of GP73 in HCV infected cells and its effect on HCV life cycle in cell culture systems. Both the protein expression and mRNA levels of GP73 significantly increased in HCV subgenomic replicon-harboring cells and HCV-infected cells, which imply that GP73 was upregulated by HCV infection. HCV production was significantly enhanced when GP73 was overexpressed, but dramatically inhibited when GP73 was silenced. However, the overexpression and knockdown of GP73 showed no evident effect on the entry, protein translation, RNA replication, and assembly of HCV, which indicates that GP73 enhanced the secretion process. Moreover, the coiled-coil domain of GP73 was required to increase HCV secretion. GP73 increased and interacted with apolipoprotein E, an identified host factor that assists in HCV secretion. These results demonstrate the critical function of GP73 in HCV secretion and provide new insights into the therapeutic design of antiviral strategies.

## Introduction

Hepatitis C virus (HCV), which is a major cause of chronic liver diseases, affects approximately 170 million people worldwide. More than 80% of infected patients develop chronic infection and one-third of these patients develop progressive liver injury, fibrosis, cirrhosis and hepatocellular carcinoma (HCC) over a period of 20 to 30 years [1]–[3]. To date, no prophylactic or therapeutic vaccine for HCV is available. The combinative administration of PEGylated-interferon(IFN)-α and ribavirin remains the standard treatment for chronic hepatitis C, but its efficiency varies across different genotypes and causes severe side effects [4]. Therefore, the development of novel HCV drug therapies is urgently needed and greatly relies on a better understanding of the life cycle of HCV.

HCV, a small enveloped RNA virus, belongs to the *Hepacivirus* genus of the *Flaviviridae* family, which also includes several classic flaviviruses, such as dengue virus and yellow fever virus [5]. Similar to other flaviviruses, the life cycle of HCV involves attachment and entry, protein translation, RNA replication, assembly and release[6]. HCV pseudoparticles (HCVpp), subgenomic replicon (SGR), and HCV cell culture systems (HCVcc) have been developed in recent years to study the mechanisms underlying the HCV life cycle [7]–[10]. Although great progress continuously being made, our knowledge of such mechanisms, particularly of virion assembly and secretion, remains highly limited [6].

Recent studies have revealed the importance of Golgi components in HCV maturation. Protein kinase D (PKD) is recruited to the trans-Golgi network to influence vesicular trafficking to the plasma membrane. The expression of HCV proteins inactivates PKD to enhance HCV secretion [11]. In addition, Golgi-localized phosphatidylinositol 4-phosphate and its interacting protein Golgi phosphoprotein 3 are required for HCV secretion [12]. A recent study used targeted siRNA screens, to identify and prove that several other Golgi-related proteins, including cytohesin-3 (responsible for Golgi structures and functions), protein kinase D1, adaptor-related protein complex 1, phosphatidylinositol 4-kinase beta and clathrin interactor 1 (responsible for cargo sorting and vesicle budding from the trans-Golgi network), are involved in HCV virion secretion [13]. Other studies also confirmed that Golgi protein 73 (GP73), also known as GOLPH2 or GOLM1, is upregulated in the liver samples of HCV infected patients [14], [15]. Moreover, the serum GP73 level is significantly higher in HCV-positive patients with HCC than that in healthy individuals [16]. GP73 is a type-II membrane protein residing in the *cis-* and *medial*- Golgi cisternae [17]. GP73 overexpression has been identified in various acute and chronic liver diseases [14], [18]–[20], and serum GP73 is considered as a better biomarker in the early diagnosis of liver diseases than the conventional alpha-fetoprotein [21]–[24]. Based on protein sequence analysis, GP73 is conserved among different species, and shares a short cytoplasmic N-terminus, a transmembrane domain (TMD), and a coiled-coil domain [25], [26]. Previous studies showed the following findings: GP73 exists as a dimer that is mainly mediated by the conserved coiled-coil domain, and the dimer is stabilized by two inter-protein disulfide bonds formed by two conserved cysteines in the coiled-coil domain; the Golgi localization of GP73 is determined by the TMD and a single positively charged residue flanking the TMD on the cytoplasmic site; GP73 interacts with other cellular proteins, including clusterin, mainly through the coiled-coil domain [25], [27]. However, the exact function and biological function of GP73 in HCV infection have not been investigated at the cellular level.

To determine the correlation between GP73 and HCV infection at the cellular level, the effect of GP73 on the HCV life cycle and its regulation in HCV-infected cells were examined in this study. Our results suggest that GP73 was upregulated by HCV infection, and GP73 could enhance HCV virion secretion by increasing apolipoprotein E (APOE) and interacting with APOE. Our results confirm the connection between GP73 and HCV infection at the molecular and cellular levels for the first time, and provide new insights into the therapeutic design of anti-HCV strategies.

## Materials and Methods

### Plasmid construction

*GP73* truncation expression plasmids were generated as previously described [25]. Plasmid pJc1 (APP23) containing the full-length Jc1 HCV sequence was provided by Apath L.L.C (USA). Plasmid pJc1-GFP, which was used to produce HCV-GFP virus, was constructed by inserting the coding sequence of green fluorescence protein (GFP) in the NS5A gene as previously described [28]. HCV SGR pSGR-JFH1 was provided by Dr. Jin Zhong (Institute Pasteur of Shanghai, Chinese Academy of Sciences) and pSGR-Con1 was constructed as previously described [7]. Plasmid pRLenti-GP73 was constructed by subcloning the open reading frame of *GP73* into the lentiviral vector pRlenti. Plasmids pSuper-shGP73-1 and pSuper-shGP73-2, which were used to generate siRNA against *GP73*, were constructed by inserting the target sequence into the pSuper (Oligoengine, USA) in site with Bgl II and Hind III. The inserted sequences are as follows:

**shGP73-1:5'-**GATCCCCGTTGAGAAAGAGGAAACCAATTTCAAGAGAATTGGTTTCCTCTTTCTCAACTTTTTA-3**'**.

**shGP73-2:5'-**GATCCCCCGAATAGAAGAGGTCACCAAATTCAAGAGATTTGGTGACCTCTTCTATTCGTTTTTA-3'.

The target sequences against GP73 are underlined. The glycoprotein sequences of HCV J6 and JFH1 were cloned into pCDNA3.1 to generate pJ6E1E2 and pJFH1E1E2 for HCVpp production as previously reported [29].

### Cells and reagents

Huh7, Huh7.5.1, and HEK 293T cells were cultured in Dulbecco’s modified Eagle’s medium (Gibco, USA) supplemented with 10% fetal bovine serum (Hyclone, USA), 100 units/mL penicillin, and 0.1% (w/v) streptomycin. pSGR-Con1 and pSGR-JFH1 RNA were transfected into Huh7 and Huh7.5.1 cells, respectively. HCV SGR-harboring cells (Huh7-SGR and Huh7.5.1-SGR) were subsequently established as previously described [7]. Huh7-SGR cured cells and Huh7.5.1-SGR cured cells were obtained by treating SGR cells with 270 ng/mL of pegylated interferon α-2a (pegasys) for 2 weeks. Huh7.5.1 cells were infected with the GP73 and shGP73 lentiviral particles and cultured in the medium supplemented with 800 µg/mL G418 and 4 µg/mL puromycin, respectively. After three weeks of selection, the surviving cells were pooled and designated as Huh7.5.1 GP73 and Huh7.5.1 shGP73-1.

HCV core monoclonal antibody (C7-50) and NS3 monoclonal antibody (H23) were purchased from Abcam (USA). Anti-FLAG monoclonal antibody M2 was purchased from Sigma (USA). Rabbit polyclonal antibody anti-APOE was purchased from Sino Biological Inc. (Beijing, China). Anti-GP73 monoclonal antibody (5B12) and rabbit polyclonal antibody were prepared in our laboratory.

### HCV-GFP virus preparation and titration

The *in vitro* RNA transcription, transfection into Huh7.5.1, and preparation of HCV-GFP viruses were all performed as previously reported [30], [31]. For titration, the viral supernatant was centrifuged to remove cell debris and serially diluted to infect Huh7.5.1 cells. After 72 h, the cells were fixed with 4% paraformaldehyde for observation under fluorescence microscopy. Virus titers were calculated as focus forming units per milliliter.

### HCVpp preparation and infection

HCVpp was prepared as previously reported [29]. The target cells were incubated with a virus-containing supernatant in the presence of 4 µg/mL polybrene (Sigma, USA) for 6 h. Infection efficiency was measured by a flow cytometer (Accrui C6, BD Biosciences) 72 h after infection.

### Lentiviral infection

To produce infectious GP73 and shGP73 lentiviral particles, HEK293T cells were co-transfected with either lentiviral vector pRLenti-GP73 or pSuper-shGP73-1/2 together with packaging plasmid psPAX2 (Addgene plasmid 12260) and pMD2.G (Addgene plasmid 12259). At 48 h after transduction, the virus-containing supernatant was filtered through 0.45 µm filters, and incubated with the target cells in the presence of 4 µg/mL polybrene (Sigma, USA).

### Immunofluorescence assay

Cells were seeded onto cover slides 24 h after transfection or 48 h after infection. After overnight culture, slides were directly fixed in 4% paraformaldehyde in PBS for 15 min, followed by permeabilization with 0.5% Triton X-100 for 5 min. These cells were then stained in diluted antibodies. The FLAG epitope in the fusion proteins was detected using anti-FLAG monoclonal antibody M2 (Sigma, USA), followed by goat anti-mouse immunoglobulin conjugated to Alexa Fluor 488 dye (Molecular Probes, USA). APOE was detected using anti-APOE rabbit polyclonal antibody and a goat anti-rabbit immunoglobulin conjugated to Alexa Fluor 488 dye (Molecular Probes, USA). Endogenous GP73 was detected using anti-GP73 monoclonal antibody (5B12) or rabbit polyclonal antibody prepared in our laboratory. The slides were viewed and imaged using a confocal microscope (LSM 710, Carl Zeiss).

### Sucrose density gradient centrifugation

The culture medium of HCV-infected cells was centrifuged to remove cellular debris and filtered through 0.45 µm filters. The supernatant was pelleted by centrifugation at 100,000 ×g for 3 h at 4°C. The pellet was resuspended in 400 µL PBS buffer and applied on a 20%–60% sucrose gradient (3.5 mL volume) in SW60 tubes (Beckman Coulter) and centrifuged at 100,000 × g (RCFav) for 16 h at 4°C. We collected 340 µL fractions from the top of the gradient. The fractions were tested for protein levels using western blot and relative viral titer with a flow cytometer.

### Co-immunoprecipitation (co-IP) assay

For intracellular GP73 and APOE interaction, 293T cells were cotransfected with plasmids expressing FLAG-tagged GP73 and HA-tagged APOE. At 30 h after transfection, cells were harvested and lysed in freshly prepared immunoprecipitation buffer consisting of 50 mM Tris–HCl (pH 7.4), 150 mM NaCl, 5 mM EDTA, 0.5% NP40 and protease inhibitor cocktail (Sigma) for 20 min on ice, followed by centrifugation at 16,000 ×g for 30 min at 4 °C. The soluble supernatants were processed for immunoprecipitation by incubation with specific antibodies or normal mouse IgG control overnight at 4 °C. Protein A/G beads (Amersham Bioscience) were added for an additional 3 h. The immunoprecipitants were washed eight times with PBS. Associated proteins were resolved by SDS-PAGE and immunoblotted with the indicated antibodies.

For secreted GP73 and APOE interaction, the culture medium was collected 48 h after transfection and centrifuged to remove cellular debris. The supernatants were concentrated by an Amicon Ultra -15 centrifugal filter (Millipore, USA) and processed for immunoprecipitation as described above.

### Quantitative Reverse Transcription PCR (qRT-PCR)

The total RNA was extracted from the cells using a Total RNA Isolation Kit (Sangon Biotech, China) or from the supernatants using QIAamp Viral RNA Mini Kit (QIAGEN, Hilden, Germany) according to the manufacturer’s instructions. First-strand cDNA was synthesized using RevertAid reverse transcriptase (Fermentas, USA) and qRT-PCR was performed using Premix Ex Taq (Takara, Japan) in a CFX96 Real-Time PCR Detection System (Bio-rad, USA). The primers used in this study are listed in Table S1.

### Intracellular and supernatant HCV-GFP titration

For intercellular HCV-GFP, the infected cells were harvested and lysed by four freeze-thaw cycles in –80°C and 37°C water baths. Cell debris was precipitated by centrifugation at 4000 rpm for 5 min and the supernatant was collected for intracellular HCV titration. For the supernatant HCV-GFP, the medium of the infected cell was collected at the indicated time points and centrifuged to remove the cell debris for supernatant HCV titration. The supernatant from either intracellular or supernatant HCV was incubated with 5×10^4^ naive Huh7.5.1 in a 24-well plate for 6 h. At 72 h post-infection, the infected cells were detected using a flow cytometer (Accrui C6, BD Biosciences). The intracellular and supernatant HCV titers were represented as relative titers calculated as percentages of the GFP-positive rate of the control, which corresponded to the absolute HCV titer (Figure S1).

### Statistical analysis

Data are shown as means ± standard error of the mean (SEM). Statistical analysis was performed using GraphPad Prism software (GraphPad, San Diego, CA, USA). P<0.05 was considered to be statistically significant.

## Results

### GP73 is upregulated by HCV infection

**Figure 1:**
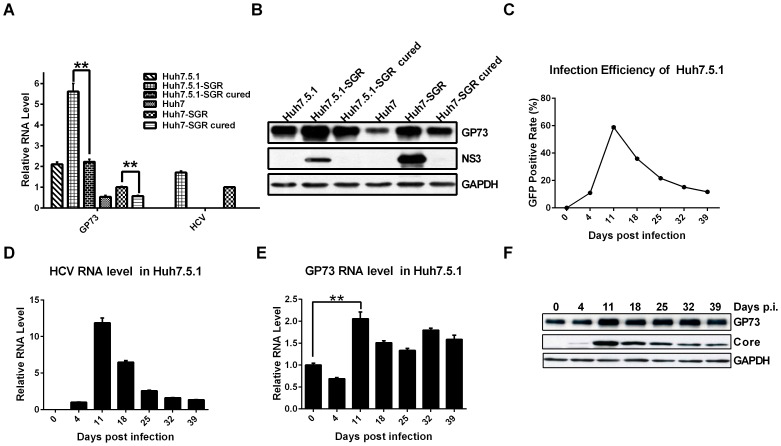
GP73 is upregulated by HCV infection. (**A**) GP73 mRNA and viral RNA in SGR-harboring and cured cells. The values are represented relative to those of Huh7-SGR. (**B**) Western blot of GP73 and HCV NS3 proteins in HCV SGR-harboring and cured cells. (**C**) Huh7.5.1 cells were infected with HCV-GFP at 0.02 MOI. Infection efficiency was detected using a flow cytometer at the indicated time points. (**D**) Intracellular HCV RNA levels were quantified by qRT-PCR at the indicated time points. The values are represented relative to those of 4 d post-infection. (**E**) The mRNA level of GP73. The values are represented relative to those of the control. (**F**) The expression level of GP73 protein and HCV core protein. The results are presented as mean ± SEM derived from three experiments (*P<0.05; **P<0.01).

Previous studies reported the enhanced GP73 expression in the liver and sera of HCV-positive patients with cirrhosis or HCC [14], [16]. To determine whether GP73 is augmented by HCV replication in the cell culture system, HCV SGR-harboring cells were established by transfecting HCV SGR replicon into Huh7 or Huh7.5.1, followed by the examination of GP73 expression. Both the mRNA and protein expression levels of GP73 were significantly upregulated in HCV SGR-harboring cells and declined when the HCV genome was cleared by treatment of IFN-α **(** Figures 1A and 1B). To investigate whether GP73 is upregulated by HCV infection, Huh7.5.1 cells were infected with HCV-GFP virus, and GP73 expression was detected at the indicated time points. Both the efficiency of HCV infection and quantity of viral RNA increased, peaked 11 d after infection, and decreased thereafter (Figures 1C and 1D). Similarly, the mRNA and protein expression levels of GP73 were also significantly upregulated and peaked 11 d after infection **(** Figures 1E and 1F). These results suggest that GP73 is upregulated by HCV infection.

### GP73 significantly enhances HCV production

**Figure 2:**
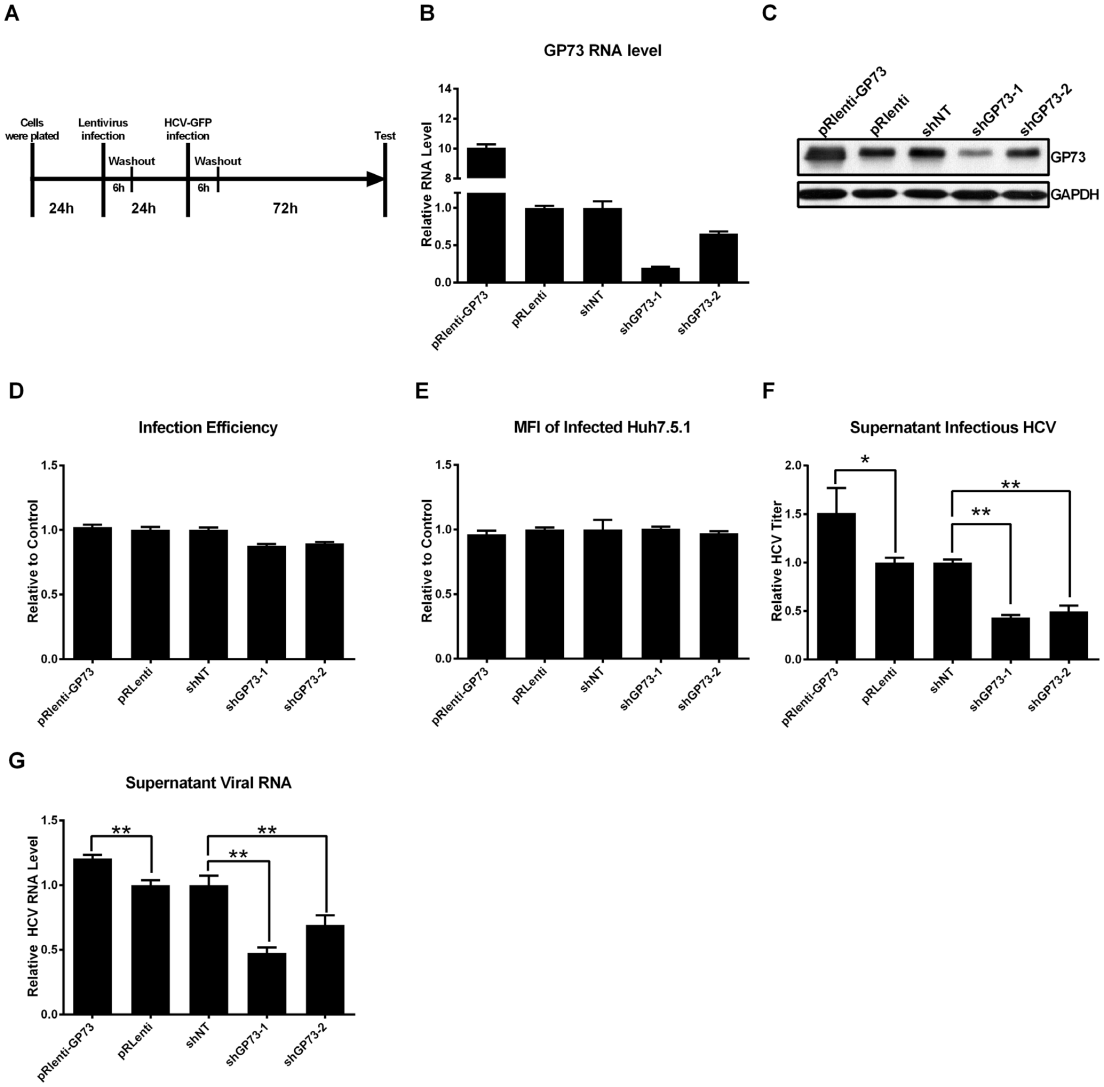
GP73 increases HCV production in the supernatant. (**A**) Schematic of the experimental procedure. Huh7.5.1 cells were infected with the lentivirus carrying the coding sequence of GP73 (pRlenti-GP73), vector control (pRlenti), shRNA expression cassette expressing shRNA against GP73 (shGP73-1 and shGP73-2), or non-target control (shNT) for 6 h. After 24 h, Huh7.5.1 cells were infected with HCV-GFP at 0.02 MOI for another 6 h. The GP73 mRNA levels (**B**), GP73 protein levels (**C**), the infection efficiency (**D**), and MFI (**E**) of Huh7.5.1 cells were assayed 72 h after infection with HCV-GFP. The HCV-GFP titer (**F**) and HCV viral RNA levels (**G**) in the supernatant were assayed by flow cytometry or qRT-PCR. The value of pRlenti-GP73 is represented relative to that of pRlenti. The values of shRNAs against GP73 are represented relative to those of shNT. The results are presented as mean ± SEM derived from three experiments (*P<0.05; **P<0.01).

To determine the influence of GP73 on HCV infection, we investigated whether GP73 participated in HCV entry, viral RNA replication, and the production of infectious HCV in the culture supernatant. Different cell-based assays were used to address these questions. First, GP73 was overexpressed or silenced in Huh7.5.1 cells through the lentiviral delivery system (Figure 2A). The mRNA and protein levels of GP73 were analyzed (Figures 2B and 2C). No significant difference was observed in the infection efficiency (Figure 2D) regardless of the expression levels of GP73. Moreover, no significant difference in the mean fluorescent intensity (MFI) was observed, which indicates that GP73 did not influence the protein expression level of NS5A-GFP of HCV (Figure 2E). However, as shown in Figure 2F, the overexpression of GP73 significantly increased the supernatant infectious HCV (pRLenti-GP73), whereas the inhibition of GP73 markedly decreased the supernatant infectious HCV (shGP73-1, shGP73-2). The corresponding changes in the supernatant HCV RNA level were also observed (Figure 2G).

**Figure 3:**
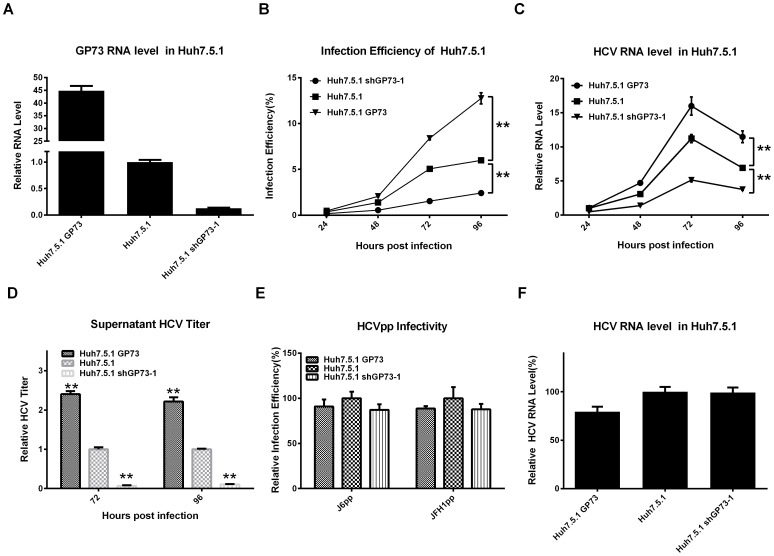
GP73 enhances HCV production. (**A**)The mRNA levels of GP73 in stable cell lines were detected by qRT-PCR. (**B**) Infection efficiency of HCV-GFP in Huh7.5.1 stable cells was detected with flow cytometry at the indicated time points after infection at 0.02 MOI. (**C**) Intracellular viral RNA in Huh7.5.1 cells was quantified by qRT-PCR. The values are normalized to those of 24 h post-infection. (**D)** HCV-GFP infectivity of the supernatant at 72 h and 96 h post-infection were assayed by flow cytometry. (**E**) HCVpp infectivity in stable cells. The values are represented relative to naive Huh7.5.1 after normalization with VSVGpp infectivity. (**F**) HCV RNA levels in stable cells at 96 h post-transfection with pSGR-JFH1. The values are represented relative to those of naive Huh7.5.1 after normalization with HCV RNA level at 6 h post-transfection. The results are presented as mean ± SEM derived from three experiments (*P<0.05; **P<0.01).

In the second system, stable Huh7.5.1 cell lines were established, in which GP73 was either overexpressed using a GP73-expressing lentiviral vector or knocked down using a shGP73-expressing lentiviral vector (Figure 3A). These cell lines remained normal viability and morphology (data not shown). After these stable cell lines were infected with HCV-GFP at 0.02 MOI, the infection efficiency, intracellular HCV RNA, and supernatant HCV titer were measured every 24 h. In the GP73-overexpressed Huh7.5.1, the infection efficiency and intracellular HCV RNA level were markedly higher than those in the naive Huh7.5.1. However, these values were significantly lower in the GP73 knockdown cells (Figures 3B and 3C). Moreover, the overexpression of GP73 significantly increased the supernatant infectious HCV, whereas the inhibition of GP73 markedly decreased the supernatant infectious HCV (Figure 3D). Similar results were observed when the experiments were performed in Huh7 cells (Figure S2).

To investigate exclusively the effect of GP73 on HCV entry process, we measured HCVpp infectivity in the aforementioned stable cells. HCVpp infectivity did not display an evident difference among these three cell lines (Figure 3E). Moreover, to determine the influence of GP73 on viral RNA replication, an HCV SGR, pSGR-JFH1, was transfected into the three cell lines, and the viral RNA levels were detected using qRT-PCR 96 h after transfection. The results showed a slight difference in viral RNA replication level, regardless whether GP73 was overexpressed or knocked down (Figure 3F).

In summary, these results suggest that GP73 increased HCV production by enhancing its assembly and secretion process, not by its entry and RNA replication process.

### GP73 enhances HCV secretion through its coiled-coil domain

**Figure 4:**
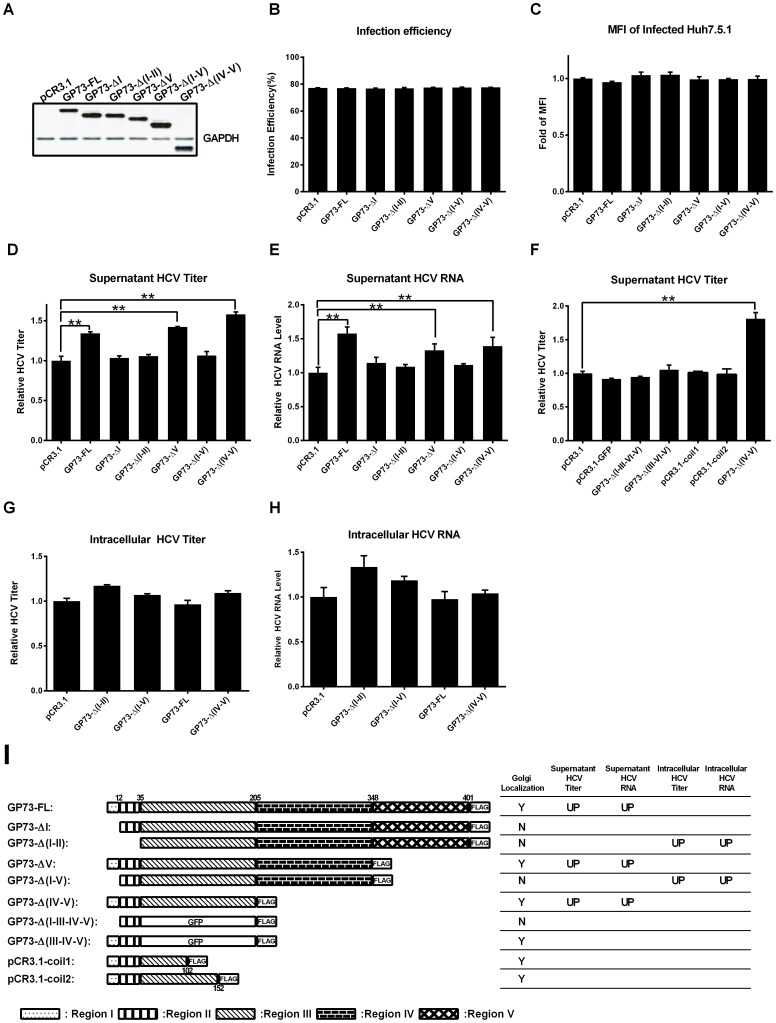
GP73 increases the secretion of HCV through the coiled-coil domain. Huh7.5.1 cells infected with HCV-GFP at 0.02 MOI were transfected with GP73 truncation expression plasmids at 48 h post-infection. At 48 h after transfection, Huh7.5.1 cells were harvested and assayed. (**A**) Protein expression levels of GP73 truncations were detected by western blot. Infection efficiency (**B**) and MFI (**C**) of HCV-infected Huh7.5.1 were assayed by flow cytometry (**D**) Supernatant HCV-GFP titer was assayed by flow cytometry. (**E**) HCV viral RNA in the supernatant was quantified by qRT-PCR. (**F**) HCV-GFP titer of the supernatant from Huh7.5.1 cells transfected with indicated plasmids was assayed by flow cytometry. (**G**) Intracellular HCV-GFP infectivity was assayed by flow cytometry. (**H**) Intracellular HCV RNA levels were quantified by qRT-PCR. (**I**) Diagram of GP73 truncation structure and summary of effects on HCV production. (Y: Yes; N: No; UP: Upregulated). The results are presented as mean ± SEM derived from three experiments (*P<0.05; **P<0.01).

In our previous study, we generated a panel of GP73 deletion mutants, allowing us to perform a detailed structure-function analysis of GP73 [25]. To ascertain the structural determinants of GP73 responsible for the enhancement of HCV production, GP73 truncation expression plasmids were transfected into Huh7.5.1 cells which were infected with HCV-GFP 48 h in advance (Figure 4A). HCV production in the supernatant was then analyzed. Similar infection efficiency (Figure 4B) and viral protein expression (Figure 4C) were observed in all truncations. However, a significant enhancement in the infectious HCV and HCV RNA in the supernatant was observed in the GP73-FL, GP73-ΔV, and GP73-Δ(IV-V) transfected cells , but not in the GP73-ΔI, GP73-Δ(I-II), and GP73-Δ(I-V) transfected cells (Figures 4D and 4E). These results suggested that the cytoplasmic tail (region I), the TMD (region II), and coiled-coil domain (region III) of GP73 were required to increase the supernatant HCV production. We previously found that regions I and II of GP73 are required for its Golgi localization, indicating that Golgi localization of GP73 is necessary in the GP73 mediated enhancement of the supernatant HCV. To determine how region III contributes to the increase in supernatant HCV, we examined the effect of two additional GP73 mutant proteins on the supernatant HCV production. GP73-Δ(III-IV-V) was generated by replacing region III of GP73-Δ(IV-V) by GFP, and GP73-Δ(I-III-IV-V) was generated by deleting region I of GP73-Δ(III-IV-V) [25]. The loss of region III diminished the enhancement of supernatant HCV production, which further confirmed that region III of GP73 was necessary in the enhancement of the supernatant HCV (Figure 4F). Region III of GP73 was predicted to contain three α-helix segments (56–102aa, 103–152aa and 153–205aa) [25]. To determine which segment of region III dominates the supernatant HCV production, two other GP73 truncation mutants were constructed, which contained regions I, region II, and the first α-helix segment and region I, region II, and the first two α-helix segments, respectively. The results showed that neither the first nor the first two α-helix segments were sufficient to increase the supernatant HCV, suggesting that the complete coiled-coil domain is necessary (Figure 4F).

To illuminate whether GP73 enhances the supernatant HCV through increasing virus assembly or promoting virion release, the intracellular HCV titer and intracellular HCV RNA levels were measured after transfecting different GP73 truncation mutants into HCV-infected cells. GP73-FL and GP73-Δ (IV-V) did not increase intracellular HCV titer or viral RNA (Figures 4G and 4H), which suggests that GP73 enhanced HCV production by assisting HCV secretion, not by promoting assembly. As summarized in Figure 4I, GP73 enhanced HCV production by assisting secretion through its coiled-coil domain.

### GP73 upregulates APOE and interacts with intracellular and secreted APOE

**Figure 5:**
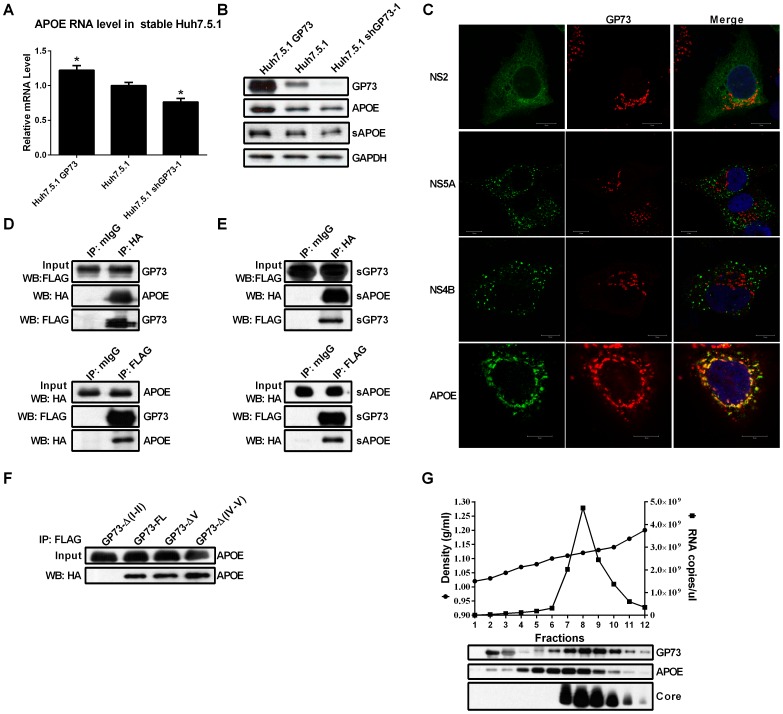
GP73 upregulates APOE and interacts with APOE. (**A**) APOE mRNA levels in stable Huh7.5.1 cells were quantified using qRT-PCR. The results are presented as mean ± SEM derived from three experiments (*P<0.05). (**B**) Protein levels of intracellular GP73, APOE, and secreted APOE (sAPOE) in stable Huh7.5.1 cells were detected by western blot. (**C**) Colocalization analysis of endogenous GP73 and HCV proteins and APOE. For NS5A and APOE, Huh7.5.1 cells were infected with HCV-GFP at 0.02 MOI. The subcellular localizations of GP73, NS5A-GFP and APOE were imaged with indirect immunofluorescence assay. For NS2 and NS4B, Huh7.5.1 cells were transfected with plasmids expressing GFP-tagged NS2 or FLAG-tagged NS4B. The subcellular localizations of GP73, NS2-GFP and NS4B were imaged with indirect immunofluorescence assay. (Bar, 10 µm.) (**D**) Co-immunoprecipitation analysis of 293T cells cotransfected with plasmids expressing FLAG-tagged GP73 and HA-tagged APOE. Cell lysates were immunoprecipitated with normal mouse IgG (mIgG), anti-Flag and anti-HA, respectively. The cell lysates and immunoprecipitates were analyzed by immunoblotting with antibodies as indicated. (**E**) Co-immunoprecipitation analysis of secreted GP73 (sGP73) and secreted APOE (sAPOE) in the culture supernatants of Huh7.5.1 cells cotransfected with plasmids expressing FLAG-tagged GP73 and HA-tagged APOE. The supernatants were immunoprecipitated with normal mouse IgG (mIgG), anti-Flag and anti-HA, respectively. The supernatants and immunoprecipitates were analyzed by immunoblotting with antibodies as indicated. (**F**) Co-immunoprecipitation analysis of 293T cells cotransfected with plasmids expressing FLAG-tagged GP73 truncations and HA-tagged APOE. Cell lysates were immunoprecipitated anti-Flag antibody. The cell lysates and immunoprecipitates were analyzed by immunoblotting with anti-HA antibodies as indicated. (**G**) Sucrose density gradient analysis of the culture medium of HCV-GFP infected cells. (*Upper*) Supernatant from infected Huh-7.5.1 cells was fractionated as described in *Materials and Methods*. The buoyant density of sucrose is plotted with the viral RNA of HCV-GFP measured by qRT-PCR. (*Lower)* Western blot analysis of GP73, APOE, and core proteins in the fractions of sucrose gradient.

Many new host cofactors required in HCV egress were recently identified[13]. To investigate the possible mechanism of GP73 on HCV secretion, we first analyzed the expressions levels of these cofactors in stable Huh7.5.1 cells with different levels of GP73 expression. As shown in Figure 5A, APOE mRNA level increased in Huh7.5.1 GP73 cells, but decreased in GP73 shGP73-1 cells. Consistent with these findings, a pronounced increase in intracellular and secreted APOE protein levels in Huh7.5.1 GP73 cells and a decrease in intracellular and secreted APOE protein levels in Huh7.5.1 shGP73-1 cells were also observed (Figure 5B). When the subcellular localizations of GP73, HCV viral proteins (NS2, NS4B, and NS5A), and APOE were detected, no obvious colocalization between GP73 and HCV proteins was observed. By contrast, GP73 and APOE colocalized in the Golgi apparatus (Figure 5C).

To determine whether GP73 and APOE interact directly, co-IP assays were performed. As indicated by Figure 5D, the intracellular GP73 and APOE formed complexes. Given that both GP73 and APOE can be secreted, we found that both proteins also existed as a complex in their secreted form (Figure 5E). As shown in Figure 5F, GP73-FL, GP73-ΔV and GP73-Δ(IV-V), all of which enhanced the secretion of HCV virion, could interact with APOE. However, GP73-Δ(I-II) (the GP73 variant faild to enhance the secretion of HCV) could not interact with APOE. APOE, a key component of HCV virion, is required for HCV infectivity [32]–[34]. To determine if GP73 is also associated with infectious HCV particles, HCVcc was fractionized using sucrose density gradient centrifugation. Subsequently, the buoyant density, HCV viral RNA and protein levels of GP73, APOE, and HCV core in each fraction were detected. GP73, APOE and core were simultaneously detected in the fractions with high HCV RNA level (the fractions 7, 8, and 9 in Figure 5G). The results indicate that GP73 is associated with HCV virion.

The overall results suggest that one underlying mechanism of GP73 enhanced HCV production is through the upregulation of APOE and its assistance in HCV secretion through interaction with APOE.

## Discussion

Serum GP73 is considered as a biomarker for liver diseases with clinically proven sensitivity and specificity [19], [35]. Several recent studies indicate that HCV infection is correlated with upregulated GP73 in clinical samples [14]–[16]. However, little is known about the physiological functions and regulatory mechanism of GP73 during HCV infection.

To investigate whether HCV infection upregulates GP73 expression at the cellular level, we detected the change in expression of GP73 in HCV SGR-harboring cells and HCV-infected cells. The mRNA and protein expression levels of GP73 significantly increased in SGR-harboring cells and declined when the HCV genome was cleared, either in Huh7 or Huh7.5.1 cells (Figures 1A and 1B). The expression of GP73 in Huh7.5.1 cells was higher than that in Huh7 cells. One probable reason is that Huh7.5.1 cells were derived from the Huh7.5 cells with clearance of harboring HCV replicon by human IFN-γ [36]. In addition, Huh7.5 cells, a cell line with more capability of supporting HCV infection than Huh7, were derived from the Huh7 cells with clearance of harboring HCV replicon by human IFN-α [37]. The higher GP73 expression level in Huh7.5.1 cells possibly suggests the important function of GP73 in effectively supporting HCV infection, and partly accounts for the different degrees of upregulation of GP73 by HCV replicon in the two cell lines (Figures 1A and 1B).

The upregulation of GP73 in SGR-harboring cells indicates that GP73 was probably upregulated by viral RNA replication and non-structural protein expression. However, no evident change in the expression of GP73 was observed in stably cells expressing non-structural proteins (Figure S3). This finding minimizes the probability that GP73 was regulated by the expression of one specific non-structural protein. In persistent HCV-infected cells, the viral replication pattern of rapidly peaking followed by a slow decline was similar to the pattern of HCV acute infection and subsequent chronic infection in clinical patients [38] (Figures 1C and 1D). Meanwhile, GP73 was remarkably upregulated in late HCV-infected cells (10 d after infection) but not in early HCV-infected cells (4 d after infection) (Figure 1). Previous results revealed the existence of co-evolutionary events in both host and virus during persistent HCV infection [39]. Based on the aforementioned results, we hypothesize that the upregulation of GP73, which assists the virus in efficiently releasing virions, is resulted from the transformation of cells caused by persistent HCV infection. However, further investigations are needed to verify this hypothesis and disclose its underlying mechanism.

Recent studies suggest the involvement of the Golgi apparatus in HCV secretion [11]–[13], [40]. Our study extended this knowledge to another resident Golgi protein, GP73. The overexpression of GP73 significantly enhanced HCV secretion without any influence on HCV entry and RNA replication in Huh7.5.1 cells. Moreover, the severe suppression of HCV secretion by knocked-down of GP73 expression reveals the potential of GP73 as a target for anti-HCV drug development (Figure 3). The coiled-coil domain of GP73 and its Golgi localization are necessary for the enhancement of HCV production (Figure 4). Our previous study showed that the coiled-coil domain of GP73 is highly conserved, and serves as the interaction domain for the homodimerization of GP73 and its interaction with other proteins such as clusterin [25], [27]. These results suggest that GP73 possibly recruits other proteins in HCV secretion through the coiled-coil domain.

APOE, a previously defined HCV secretion related gene, was upregulated by GP73. The increase in the intracellular and supernatant APOE in GP73-overexpressed cells indicates that the upregulation of GP73 enhanced the expression and secretion of APOE (Figure 5B). Recent studies suggest intracellular APOE facilitates lipid recruitment at different stages of very low-density lipoprotein (VLDL) assembly and trafficking through the endoplasmic reticulum-Golgi secretory compartments [41]. To date, mounting evidence indicated that HCV exploits the hosts’ pathway of producing VLDLs to assemble and secrete virions [42]–[45]. One possibility is that GP73 enhances HCV secretion through increasing the assembly and secretion of VLDL by upregulating intracellular APOE. The effect of GP73 expression on VLDL assembly and secretion needs further investigations. Meanwhile, the interaction between GP73 and APOE in intracellular and secreted conditions suggests that GP73 and APOE may be secreted together through the similar secretory pathway (Figures 5D and 5E). The release of infectious HCV into the culture medium depends on the secretion of APOE [34]. Thus, another possibility is that GP73 enhances HCV secretion by increasing the secretion of APOE to facilitate the release of HCV virion. The detection of GP73 together with APOE and HCV core protein in the fractions with high HCV infectivity suggests the possible association of GP73, APOE, and HCV virion (Figure 5G). Previous studies showed that the interaction between APOE and NS5A is important for the release of infectious viral particles [46], [47]. However, we failed to identify the interaction between GP73 and NS5A through co-IP assay (data not shown). If the possibility of insufficient detection sensitivity of antibody can be ruled out, the results indicate that the association among GP73, APOE, and HCV virion is not accomplished through a similar pathway. APOE has been shown to facilitate the entry of HCV through its interaction with low-density lipoprotein receptor [48]. However, the lack of effect of GP73 expression on HCVpp infectivity and the inability of the GP73 antibody to block HCV infection both indicate that the association of GP73 with HCV virion does not participate in the entry process of HCV (Figure 3E, and data not shown). Furthermore, not thoroughgoing decrease of APOE in GP73 knocked-down cells implies the existence of other pathways, besides APOE, in GP73 to enhance HCV secretion.

APOE allele epsilon 4 is closely related to Alzheimer’s disease [49], [50]. Genome-wide association studies showed that two single-nucleotide polymorphisms in GP73, rs10868366 and rs7019241, are associated with the pathogenesis of Alzheimer’s disease [51]–[54]. The interaction between GP73 and APOE gives a hint to understand the role of GP73 in the pathogenesis of Alzheimer’s disease. Investigation of the interaction between GP73 and APOE will advanced our understanding of the physiological significance of GP73, a protein with unknown functions.

In summary, we demonstrate that GP73, a novel biomarker for liver diseases, is upregulated by HCV infection and enhances HCV secretion. In addition, we illustrate the possible mechanism by which GP73 increases HCV secretion. APOE, an identified host factor that facilitates HCV secretion, is upregulated by GP73, and can interact with GP73. The study is the first to confirm the correlation between GP73 and HCV infection at the cellular level. The identification of the involvement of GP73 in the process of HCV secretion strengthens the key role of the Golgi in HCV secretion and provides new insights into the therapeutic design of anti-HCV strategies and physiological function of GP73.

## Supporting Information

Figure S1 Infection efficiency measured by flow cytometry is highly coincident to the HCV titer. Naive Huh7.5.1 cells were incubated with HCV-containing supernatant at different volumes for 6 h before washout. Then, infectivity was detected by flow cytometry at 72 h post-infection.
(TIF)

Figure S2 GP73 enhances HCV production in stable Huh7 cells. Stable GP73 overexpressed cells (Huh7 GP73) and GP73 knockdown cells (Huh7 shGP73) were established with lentivirus as described in the “Materials and Methods” section. (**A**) GP73 mRNA level in stable cells. (**B**) Infection efficiency of HCV-GFP in stable Huh7 cells at 0.02 MOI. (**C**) Intracellular viral RNA level. (**D**) Supernatant infectivity at 96 h post-infection. The results were presented as mean ± the SEM.
(TIF)

Figure S3 GP73 is not upregulated by HCV non-structural protein expression. (A) Huh7 cells were transfected with indicated plasmids. GP73 mRNA level was quantified by qRT-PCR at 72 h post-transfection. (**B**) Huh7 cells that stably express HCV non-structural protein were established with lentivirus infection and puromycin screening. GP73 mRNA levels were quantified by qRT-PCR. (N.S.: not significant).
(TIF)

Table S1 Sequences of qRT-PCR primers used in this study.
(DOCX)

## References

[1] Leone N, Rizzetto M (2005) Natural history of hepatitis C virus infection: from chronic hepatitis to cirrhosis, to hepatocellular carcinoma. Minerva Gastroenterol Dietol 51: 31–46. 15756144

[2] Suzuki T, Aizaki H, Murakami K, Shoji I, Wakita T (2007) Molecular biology of hepatitis C virus. J Gastroenterol 42: 411–423. 17671755 10.1007/s00535-007-2030-3

[3] Levrero M (2006) Viral hepatitis and liver cancer: the case of hepatitis C. . Oncogene 25: 3834–3847. 16799625 10.1038/sj.onc.1209562

[4] Alexopoulou A, Papatheodoridis GV (2012) Current progress in the treatment of chronic hepatitis C. . World J Gastroenterol 18: 6060–6069. 23155334 10.3748/wjg.v18.i42.6060 PMC3496882

[5] Kato N (2001) Molecular virology of hepatitis C virus. Acta Med Okayama 55: 133–159. 11434427 10.18926/AMO/32025

[6] Suzuki T, Ishii K, Aizaki H, Wakita T (2007) Hepatitis C viral life cycle. Adv Drug Deliv Rev 59: 1200–1212. 17825945 10.1016/j.addr.2007.04.014

[7] Lohmann V, Korner F, Koch J, Herian U, Theilmann L, et al (1999) Replication of subgenomic hepatitis C virus RNAs in a hepatoma cell line. Science 285: 110–113. 10390360 10.1126/science.285.5424.110

[8] Lindenbach BD, Evans MJ, Syder AJ, Wolk B, Tellinghuisen TL, et al (2005) Complete replication of hepatitis C virus in cell culture. Science 309: 623–626. 15947137 10.1126/science.1114016

[9] Ploss A, Evans MJ (2012) Hepatitis C virus host cell entry. Curr Opin Virol 2: 14–19. 22440961 10.1016/j.coviro.2011.12.007 PMC3311996

[10] Rice CM (2011) New insights into HCV replication: potential antiviral targets. Top Antivir Med 19: 117–120. 21946389 PMC6148863

[11] Amako Y, Syed GH, Siddiqui A (2011) Protein kinase D negatively regulates hepatitis C virus secretion through phosphorylation of oxysterol-binding protein and ceramide transfer protein. J Biol Chem 286: 11265–11274. 21285358 10.1074/jbc.M110.182097 PMC3064182

[12] Bishe B, Syed GH, Field SJ, Siddiqui A (2012) Role of phosphatidylinositol 4-phosphate (PI4P) and its binding protein GOLPH3 in hepatitis C virus secretion. J Biol Chem 287: 27637–27647. 22745132 10.1074/jbc.M112.346569 PMC3431621

[13] Coller KE, Heaton NS, Berger KL, Cooper JD, Saunders JL, et al (2012) Molecular determinants and dynamics of hepatitis C virus secretion. PLoS Pathog 8: e1002466. 22241992 10.1371/journal.ppat.1002466 PMC3252379

[14] Kladney RD, Cui X, Bulla GA, Brunt EM, Fimmel CJ (2002) Expression of GP73, a resident Golgi membrane protein, in viral and nonviral liver disease. Hepatology 35: 1431–1440. 12029628 10.1053/jhep.2002.32525

[15] Iftikhar R, Kladney RD, Havlioglu N, Schmitt-Graff A, Gusmirovic I, et al (2004) Disease- and cell-specific expression of GP73 in human liver disease. Am J Gastroenterol 99: 1087–1095. 15180730 10.1111/j.1572-0241.2004.30572.x

[16] Riener MO, Stenner F, Liewen H, Soll C, Breitenstein S, et al (2009) Golgi phosphoprotein 2 (GOLPH2) expression in liver tumors and its value as a serum marker in hepatocellular carcinomas. Hepatology 49: 1602–1609. 19291786 10.1002/hep.22843

[17] Kladney RD, Bulla GA, Guo L, Mason AL, Tollefson AE, et al (2000) GP73, a novel Golgi-localized protein upregulated by viral infection. Gene 249: 53–65. 10831838 10.1016/S0378-1119(00)00136-0 PMC7127640

[18] Norton PA, Comunale MA, Krakover J, Rodemich L, Pirog N, et al (2008) N-linked glycosylation of the liver cancer biomarker GP73. J Cell Biochem 104: 136–149. 18004786 10.1002/jcb.21610 PMC4620713

[19] Marrero JA, Romano PR, Nikolaeva O, Steel L, Mehta A, et al (2005) GP73, a resident Golgi glycoprotein, is a novel serum marker for hepatocellular carcinoma. J Hepatol 43: 1007–1012. 16137783 10.1016/j.jhep.2005.05.028

[20] Gu Y, Chen W, Zhao Y, Chen L, Peng T (2009) Quantitative analysis of elevated serum Golgi protein-73 expression in patients with liver diseases. Ann Clin Biochem 46: 38–43. 19008260 10.1258/acb.2008.008088

[21] Ozkan H, Erdal H, Tutkak H, Karaeren Z, Yakut M, et al (2011) Diagnostic and prognostic validity of Golgi protein 73 in hepatocellular carcinoma. Digestion 83: 83–88. 21042019 10.1159/000320379

[22] Malaguarnera G, Giordano M, Paladina I, Berretta M, Cappellani A, et al (2010) Serum markers of hepatocellular carcinoma. Dig Dis Sci 55: 2744–2755. 20339916 10.1007/s10620-010-1184-7

[23] Gomaa AI, Khan SA, Leen EL, Waked I, Taylor-Robinson SD (2009) Diagnosis of hepatocellular carcinoma. World J Gastroenterol 15: 1301–1314. 19294759 10.3748/wjg.15.1301 PMC2658831

[24] Giannelli G, Antonaci S (2006) New frontiers in biomarkers for hepatocellular carcinoma. Dig Liver Dis 38: 854–859. 16782417 10.1016/j.dld.2006.05.007

[25] Hu L, Li L, Xie H, Gu Y, Peng T (2011) The Golgi Localization of GOLPH2 (GP73/GOLM1) Is Determined by the Transmembrane and Cytoplamic Sequences. PLoS ONE 6: e28207. 22140547 10.1371/journal.pone.0028207 PMC3226628

[26] Kim HJ, Lv D, Zhang Y, Peng T, Ma X (2012) Golgi phosphoprotein 2 in physiology and in diseases. Cell Biosci 2: 31. 22958594 10.1186/2045-3701-2-31 PMC3448521

[27] Zhou Y, Li L, Hu L, Peng T (2011) Golgi phosphoprotein 2 (GOLPH2/GP73/GOLM1) interacts with secretory clusterin. Mol Biol Rep 38: 1457–1462. 20842452 10.1007/s11033-010-0251-7

[28] Moradpour D, Evans MJ, Gosert R, Yuan Z, Blum HE, et al (2004) Insertion of green fluorescent protein into nonstructural protein 5A allows direct visualization of functional hepatitis C virus replication complexes. J Virol 78: 7400–7409. 15220413 10.1128/JVI.78.14.7400-7409.2004 PMC434129

[29] Bartosch B, Dubuisson J, Cosset FL (2003) Infectious Hepatitis C Virus Pseudo-particles Containing Functional E1-E2 Envelope Protein Complexes. Journal of Experimental Medicine 197: 633–642. 12615904 10.1084/jem.20021756 PMC2193821

[30] Wakita T, Pietschmann T, Kato T, Date T, Miyamoto M, et al (2005) Production of infectious hepatitis C virus in tissue culture from a cloned viral genome. Nat Med 11: 791–796. 15951748 10.1038/nm1268 PMC2918402

[31] Li X, Jiang H, Qu L, Yao W, Cai H, et al (2014) Hepatocyte Nuclear Factor 4alpha and Downstream Secreted Phospholipase A2 GXIIB Regulate Production of Infectious Hepatitis C Virus. J Virol 88: 612–627. 24173221 10.1128/JVI.02068-13 PMC3911757

[32] Jiang J, Luo G (2009) Apolipoprotein E but not B is required for the formation of infectious hepatitis C virus particles. J Virol 83: 12680–12691. 19793818 10.1128/JVI.01476-09 PMC2786834

[33] Chang KS, Jiang J, Cai Z, Luo G (2007) Human apolipoprotein e is required for infectivity and production of hepatitis C virus in cell culture. J Virol 81: 13783–13793. 17913825 10.1128/JVI.01091-07 PMC2168882

[34] Hishiki T, Shimizu Y, Tobita R, Sugiyama K, Ogawa K, et al (2010) Infectivity of hepatitis C virus is influenced by association with apolipoprotein E isoforms. J Virol 84: 12048–12057. 20826689 10.1128/JVI.01063-10 PMC2977863

[35] Mao Y, Yang H, Xu H, Lu X, Sang X, et al.. (2010) Golgi protein 73 (GOLPH2) is a valuable serum marker for hepatocellular carcinoma. Gut. 10.1136/gut.2010.214916 20876776

[36] Zhong J, Gastaminza P, Cheng G, Kapadia S, Kato T, et al (2005) Robust hepatitis C virus infection in vitro. Proc Natl Acad Sci U S A 102: 9294–9299. 15939869 10.1073/pnas.0503596102 PMC1166622

[37] Blight KJ, McKeating JA, Rice CM (2002) Highly Permissive Cell Lines for Subgenomic and Genomic Hepatitis C Virus RNA Replication. J Virol 76: 13001–13014. 12438626 10.1128/JVI.76.24.13001-13014.2002 PMC136668

[38] Barrera JM, Bruguera M, Ercilla MG, Gil C, Celis R, et al (1995) Persistent hepatitis C viremia after acute self-limiting posttransfusion hepatitis C. . Hepatology 21: 639–644. 7533121

[39] Zhong J, Gastaminza P, Chung J, Stamataki Z, Isogawa M, et al (2006) Persistent hepatitis C virus infection in vitro: coevolution of virus and host. J Virol 80: 11082–11093. 16956932 10.1128/JVI.01307-06 PMC1642175

[40] Bishe B, Syed G, Siddiqui A (2012) Phosphoinositides in the hepatitis C virus life cycle. Viruses 4: 2340–2358. 23202467 10.3390/v4102340 PMC3497055

[41] Sundaram M, Yao Z (2012) Intrahepatic role of exchangeable apolipoproteins in lipoprotein assembly and secretion. Arterioscler Thromb Vasc Biol 32: 1073–1078. 22517365 10.1161/ATVBAHA.111.241455

[42] Popescu CI, Dubuisson J (2010) Role of lipid metabolism in hepatitis C virus assembly and entry. Biol Cell 102: 63–74. 10.1042/BC20090125 19857204

[43] Huang H, Sun F, Owen DM, Li W, Chen Y, et al (2007) Hepatitis C virus production by human hepatocytes dependent on assembly and secretion of very low-density lipoproteins. Proc Natl Acad Sci U S A 104: 5848–5853. 17376867 10.1073/pnas.0700760104 PMC1829327

[44] Gastaminza P, Cheng G, Wieland S, Zhong J, Liao W, et al (2008) Cellular determinants of hepatitis C virus assembly, maturation, degradation, and secretion. J Virol 82: 2120–2129. 18077707 10.1128/JVI.02053-07 PMC2258938

[45] Nahmias Y, Goldwasser J, Casali M, van Poll D, Wakita T, et al (2008) Apolipoprotein B-dependent hepatitis C virus secretion is inhibited by the grapefruit flavonoid naringenin. Hepatology 47: 1437–1445. 18393287 10.1002/hep.22197 PMC4500072

[46] Benga WJ, Krieger SE, Dimitrova M, Zeisel MB, Parnot M, et al (2010) Apolipoprotein E interacts with hepatitis C virus nonstructural protein 5A and determines assembly of infectious particles. Hepatology 51: 43–53. 20014138 10.1002/hep.23278

[47] Cun W, Jiang J, Luo G (2010) The C-terminal alpha-helix domain of apolipoprotein E is required for interaction with nonstructural protein 5A and assembly of hepatitis C virus. J Virol 84: 11532–11541. 20719944 10.1128/JVI.01021-10 PMC2953147

[48] Owen DM, Huang H, Ye J, Gale M Jr (2009) Apolipoprotein E on hepatitis C virion facilitates infection through interaction with low-density lipoprotein receptor. Virology 394: 99–108. 19751943 10.1016/j.virol.2009.08.037 PMC2767442

[49] Saunders AM, Strittmatter WJ, Schmechel D, George-Hyslop PH, Pericak-Vance MA, et al (1993) Association of apolipoprotein E allele epsilon 4 with late-onset familial and sporadic Alzheimer's disease. Neurology 43: 1467–1472. 8350998 10.1212/wnl.43.8.1467

[50] Zlokovic BV (2013) Cerebrovascular effects of apolipoprotein E: implications for Alzheimer disease. JAMA Neurol 70: 440–444. 23400708 10.1001/jamaneurol.2013.2152 PMC4414030

[51] Li H, Wetten S, Li L, St Jean PL, Upmanyu R, et al (2008) Candidate single-nucleotide polymorphisms from a genomewide association study of Alzheimer disease. Arch Neurol 65: 45–53. 17998437 10.1001/archneurol.2007.3

[52] Lin K, Tang M, Han H, Guo Y, Lin Y, et al (2010) Association between the polymorphisms of CALHM1 and GOLPH2 genes and Alzheimer's disease. Psychiatr Genet 20: 190. 20592574 10.1097/YPG.0b013e32833a21cf

[53] Yuan Q, Chu C, Jia J (2012) Association studies of 19 candidate SNPs with sporadic Alzheimer's disease in the North Chinese Han population. Neurol Sci 33: 1021–1028. 22167654 10.1007/s10072-011-0881-0

[54] Inkster B, Rao AW, Ridler K, Filippini N, Whitcher B, et al (2012) Genetic variation in GOLM1 and prefrontal cortical volume in Alzheimer's disease. Neurobiol Aging 33: 457–465. 20570408 10.1016/j.neurobiolaging.2010.04.018

