# Supplementary figures and images for: GP73 Is Upregulated by Hepatitis C Virus (HCV) Infection and Enhances HCV Secretion
Source: PLoS One. 2014 Mar 7;9(3):e90553. doi: 10.1371/journal.pone.0090553 (PMC3946557; doi:10.1371/journal.pone.0090553)

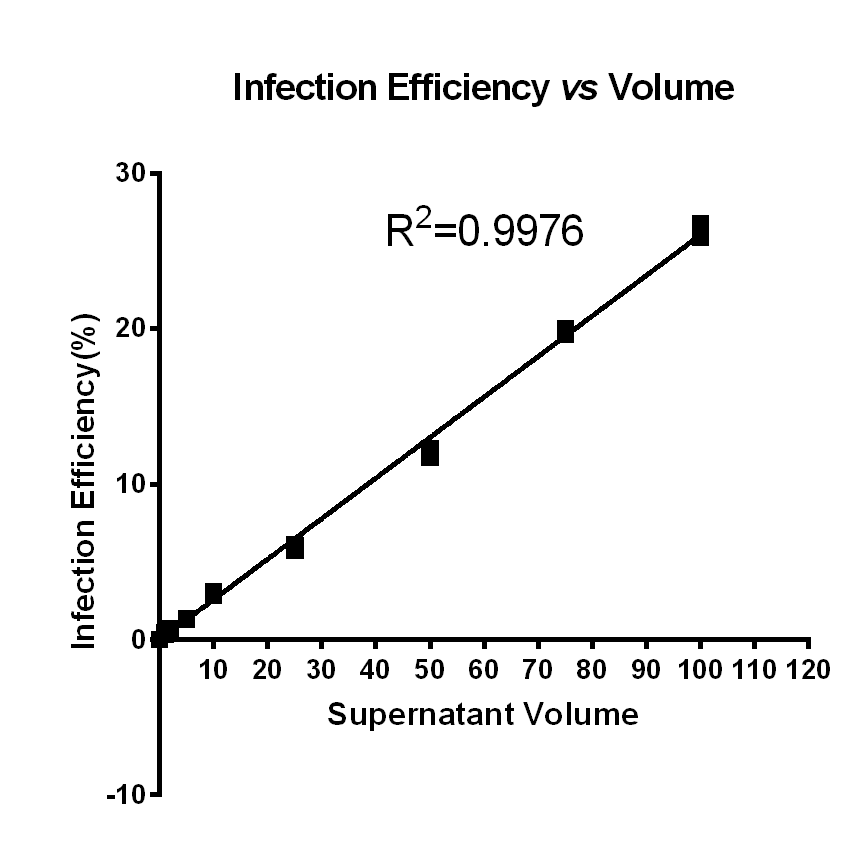

Supplement: Figure S1 — Infection efficiency measured by flow cytometry is highly coincident to the HCV titer. Naive Huh7.5.1 cells were incubated with HCV-containing supernatant at different volumes for 6 h before washout. Then, infectivity was detected by flow cytometry at 72 h post-infection. (TIF) [file pone.0090553.s001.tif]

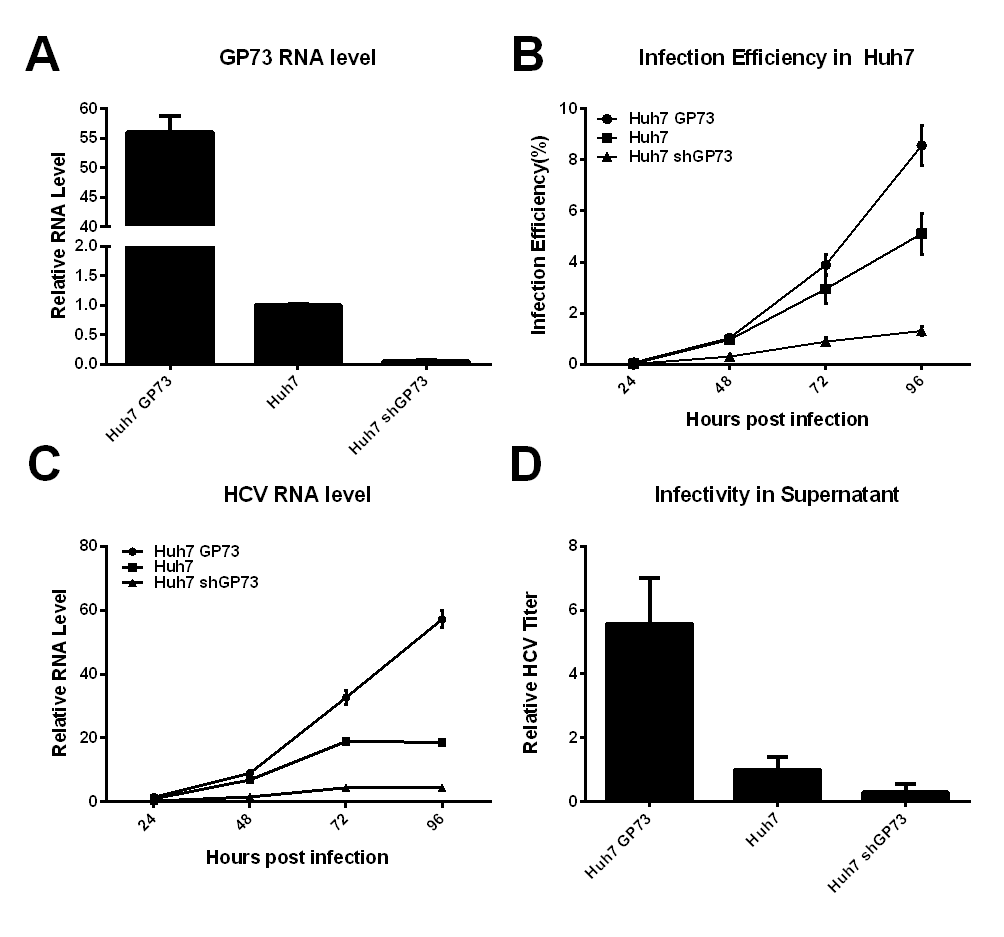

Supplement: Figure S2 — GP73 enhances HCV production in stable Huh7 cells. Stable GP73 overexpressed cells (Huh7 GP73) and GP73 knockdown cells (Huh7 shGP73) were established with lentivirus as described in the “Materials and Methods” section. (A) GP73 mRNA level in stable cells. (B) Infection efficiency of HCV-GFP in stable Huh7 cells at 0.02 MOI. (C) Intracellular viral RNA level. (D) Supernatant infectivity at 96 h post-infection. The results were presented as mean ± the SEM. (TIF) [file pone.0090553.s002.tif]

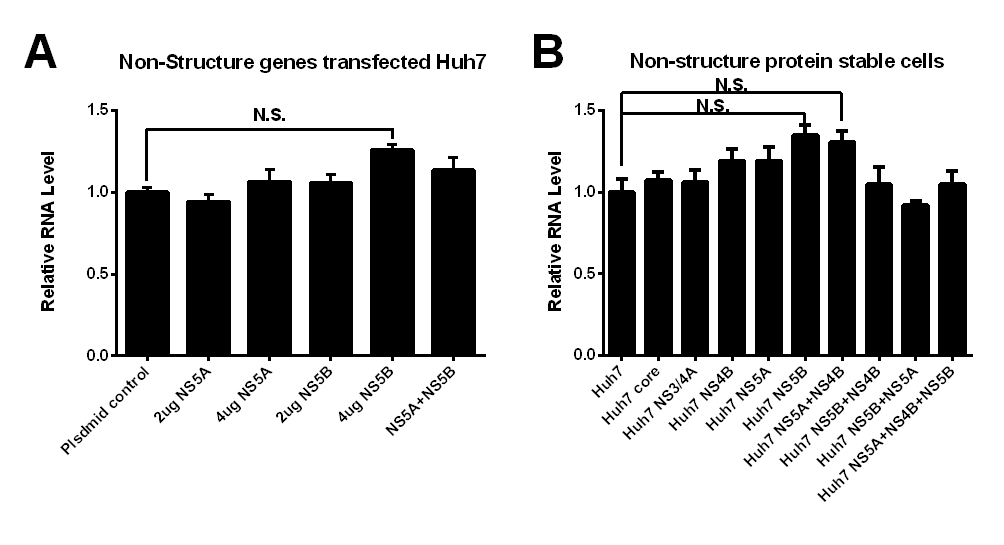

Supplement: Figure S3 — GP73 is not upregulated by HCV non-structural protein expression. (A) Huh7 cells were transfected with indicated plasmids. GP73 mRNA level was quantified by qRT-PCR at 72 h post-transfection. (B) Huh7 cells that stably express HCV non-structural protein were established with lentivirus infection and puromycin screening. GP73 mRNA levels were quantified by qRT-PCR. (N.S.: not significant). (TIF) [file pone.0090553.s003.tif]
