# Supplementary material for: GP73 Is Upregulated by Hepatitis C Virus (HCV) Infection and Enhances HCV Secretion
Source: PLoS One. 2014 Mar 7;9(3):e90553. doi: 10.1371/journal.pone.0090553 (PMC3946557; doi:10.1371/journal.pone.0090553)
Supplement: Table S1 — Sequences of qRT-PCR primers used in this study. (DOCX) [file pone.0090553.s004.docx]

Table S1

Sequences of qRT-PCR primers used in this study:

| Target | Primer sequence |
| --- | --- |
| GP73 | 5`-ACAAAATCCAGTCCAGCCAC--3` |
|  | 5`-CGTAATTCCTCTGCAGGGTC-3` |
| HCV | 5'-TCT GCG GAA CCG GTG AGT A-3' |
|  | 5'-TCA GGC AGT ACC ACA AGG C-3' |
| APOE | 5'-GCGACCGCCTGGACGAG-3' |
|  | 5'-AGGGGCTCGAACCAGCTCT-3' |
| GAPDH | 5`-CCATCTTCCAGGAGCGAGA-3` |
|  | 5`-TGGTTCACACCCATGACGAA-3` |
| 18S | 5'-GGTGAAATTCTTGGACCGGC-3' |
|  | 5'-GACTTTGGTTTCCCGGAAGC-3' |
